# Antidepressant medications reduce subcortical–cortical resting-state functional connectivity in healthy volunteers

**DOI:** 10.1016/j.neuroimage.2011.05.051

**Published:** 2011-08-15

**Authors:** Ciara McCabe, Zevic Mishor

**Affiliations:** Department of Psychiatry, Neuroscience Building, Warneford Hospital, University of Oxford, Oxford, OX3 7JXUK

**Keywords:** fMRI, Depression, Resting-state, Connectivity, Antidepressants, OFC, vmPFC

## Abstract

Studies have revealed abnormalities in resting-state functional connectivity in those with major depressive disorder specifically in areas such as the dorsal anterior cingulate, thalamus, amygdala, the pallidostriatum and subgenual cingulate. However, the effect of antidepressant medications on human brain function is less clear and the effect of these drugs on resting-state functional connectivity is unknown.

Forty volunteers matched for age and gender with no previous psychiatric history received either citalopram (SSRI; selective serotonergic reuptake inhibitor), reboxetine (SNRI; selective noradrenergic reuptake inhibitor) or placebo for 7 days in a double-blind design. Using resting-state functional magnetic resonance imaging and seed based connectivity analysis we selected the right nucleus accumbens, the right amygdala, the subgenual cingulate and the dorsal medial prefrontal cortex as seed regions. Mood and subjective experience were also measured before and after drug administration using self-report scales.

Despite no differences in mood across the three groups, we found reduced connectivity between the amygdala and the ventral medial prefrontal cortex in the citalopram group and the amygdala and the orbitofrontal cortex for the reboxetine group. We also found reduced striatal–orbitofrontal cortex connectivity in the reboxetine group.

These data suggest that antidepressant medications can decrease resting-state functional connectivity independent of mood change and in areas known to mediate reward and emotional processing in the brain. We conclude that hypothesis-driven seed based analysis of resting-state fMRI supports the proposition that antidepressant medications might work by normalising the elevated resting-state functional connectivity seen in depressed patients.

## Research highlights

► We examine the effects of 7 day treatment with two antidepressants (citalopram and reboxetine) on resting-state functional connectivity in healthy volunteers. ► Both citalopram and reboxetine reduce functional connectivity between the amygdala seed region and the prefrontal cortex. ► Antidepressant action may involve normalisation of elevated resting-state functional connectivity between key elements of resting-state networks.

## Introduction

Alterations in resting-state connectivity have been observed in depression across multiple networks. For example parts of the cognitive control network (anterior cingulate, prefrontal cortex) which are involved in decision making, attention and resolving conflicts have been found to be altered in depression (Davidson et al., 2002; Fitzgerald et al., 2006; Rogers et al., 2004; Schlosser et al., 2008; Siegle et al., 2007; Vasic et al., 2009) as has the default mode network a network involved in self-referential activity and emotional regulation (e.g. precuneus, hippocampus, medial prefrontal cortex) (Buckner et al., 2008; Grimm et al., 2011; Northoff et al.; Raichle et al., 2001; Raichle and Snyder, 2007; Sheline et al., 2009) and parts of the affective network (orbitofrontal cortex, striatum, amygdala) known to be involved in the processing of emotional and rewarding information (Johansen-Berg et al., 2008; Northoff et al.; Price and Drevets, 2009).

Recently Sheline and colleagues reported increased dorsal medial prefrontal cortex connectivity (dmPFC) in depression and suggested that differences in default mode processing may contribute to depression and thus may be a target for antidepressant drug treatment. Consistent with this idea, recent research examining emotional processing in depressed patients has also shown that dysfunctional activity in these regions is attenuated after pharmacological treatment and that this in turn correlates with treatment response (DeRubeis et al., 2005; Fu et al., 2004; Harmer et al., 2006; Hollon et al., 2005; Sheline et al., 2001; Victor et al., 2010; Wagner et al., 2010). Furthermore a recent study in depressed patients examining orbitofrontal cortex (OFC) connectivity before and after antidepressant treatment found increased OFC connectivity prior to treatment differentiated responders from non-responders (Lisiecka et al.).

Some preliminary examination of our resting-state dataset revealed decreased connectivity between the dmPFC and hippocampus following citalopram treatment compared to placebo (McCabe et al., 2011). However, it is unknown whether this is a general effect of all antidepressants or specific to those with serotonergic action. Thus we decided to investigate the dmPFC connectivity in the reboxetine group and hypothesised that unlike citalopram, reboxetine would have its effects specifically in areas where catecholaminergic pathways have a role, such as the striatum and prefrontal cortex (Eshel and Roiser; Mizoguchi et al., 2008; Valentini et al., 2004). We also decided to examine other seed regions within the affective network that we have shown in our previous studies to be modulated by reboxetine and citalopram during task-based fMRI such as the nucleus accumbens, the subgenual cingulate and the amygdala (Harmer et al., 2009, 2004; McCabe et al., 2010; Norbury et al., 2007). We investigated the effects in healthy volunteers with no history of depression or use of psychiatric medications. We hypothesised that antidepressant medications would decrease connectivity within the affective network and that this would be consistent with the recent hypotheses proposed by Sheline et al. (2010) and McCabe et al. (2011).

The time course of the current study (7 days) was limited compared to the duration of clinical treatment with antidepressants and it would therefore also be relevant to study the effect of medications on the neural basis of resting-state after longer treatment periods. However, a meta-analysis of clinical trials has shown that, relative to placebo, SSRIs significantly decrease depressive symptomatology after 1 week of treatment (Taylor et al., 2006) and indeed the effect size of active treatment relative to placebo is numerically greater during the first week of therapy than in subsequent weeks. Further, it has been demonstrated in healthy volunteers that 7 days treatment with reboxetine produces positive biases in measures of emotional perception and memory, suggesting that therapeutically relevant changes in neuropsychological function are indeed apparent during the first week of antidepressant administration (Harmer et al., 2004).

## Methods

### Participants

Forty healthy volunteers were randomised to receive 7 days oral treatment with citalopram (20 mg/day, *n* = 12), reboxetine (4 mg/b.i.d., *n* = 13) or placebo (*n* = 15), in a double blind between groups design. Medication was taken twice a day, once in the morning and once in the evening, to maintain blinding. Ethical approval was provided by the Oxford Research Ethics Committee B and written informed consent was obtained from all participants before screening and after a complete description of the study was given. Exclusion criteria for all subjects were current or past Axis-1 disorder on the Structured Clinical Interview for DSM-IV (Spitzer et al., 2004) and any contraindications to MRI e.g. pacemaker, mechanical heart valve, hip replacement, metal implants.

None of the participants took current medication apart from the contraceptive pill. Before drug administration and to ensure group matching, baseline information was collected using a neuropsychological battery that included: Beck Depression Inventory (BDI) (Beck et al., 1961), State-Trait Anxiety Inventory (STAI) (Spielberger, 1983), Fawcett–Clarke Pleasure Scale (FCPS) (Fawcett et al., 1983), and Snaith–Hamilton Pleasure Scale (SHAPS) (Snaith et al., 1995). Body mass index (BMI) was also calculated for each volunteer. To assess the effects of the treatment the following questionnaires were taken before and after the treatment: Visual Analogue Scales (VAS) of happiness, sadness, anger, disgust, alertness and anxiety; and the State Anxiety Inventory (Spielberger, 1983).

### Overall design

MRI-derived measures of brain function, based on blood-oxygenation-level-dependent (BOLD) contrast, were used to compare brain responses at rest across the three drug groups and on the 7th day approximately 5 h after the last treatment. The resting-state data were acquired after the volunteers had completed both a reward/aversion task and a structural scan (McCabe et al., 2010). Subjects were instructed to lie in dimmed light with their eyes open, think of nothing in particular, and not to fall asleep.

### fMRI scan

Images were acquired with a 3.0-T VARIAN/SIEMENS whole-body scanner at the Centre for Functional Magnetic Resonance Imaging at Oxford (FMRIB), where T2* weighted EPI slices were acquired every 2 seconds (TR = 2). Imaging parameters were selected to minimise susceptibility and distortion artefact in the orbitofrontal cortex (Wilson et al., 2002). Coronal slices (25) with in-plane resolution of 3 × 3 mm and between plane spacing of 4 mm were obtained. The matrix size was 64 × 64 and the field of view was 192 × 192 mm. Acquisition was carried out during the resting scan yielding 150 volumes in total. An anatomical T1-weighted sequence with coronal plane slice, thickness 3 mm and in-plane resolution of 1.0 × 1.0 mm was also acquired to improve the registration process.

### Analysis methods

#### Pre-processing

Data analysis was carried out using FSL tools (www.fmrib.ox.ac.uk/fsl) (Smith et al., 2004). After discarding the first four volumes to allow for T1 equilibration effects, fMRI data analysis was carried out using FEAT (FMRI Expert Analysis Tool) Version 5.98, part of FSL (FMRIB's Software Library, www.fmrib.ox.ac.uk/fsl). The pre-processing included the following steps: motion correction (Jenkinson and Smith, 2001) non-brain removal (Smith, 2002); spatial smoothing using a Gaussian kernel of full-width at half maximum (FWHM) of 6.0 mm; grand-mean intensity normalisation of the entire 4D dataset by a single multiplicative factor and high pass temporal filtering (Gaussian-weighted least-squares straight line fitting, with sigma = 75.0 s). fMRI volumes were registered to the individual's structural scan and standard space images using FMRIB's Linear Image Registration Tool (FLIRT) (Jenkinson et al., 2002; Jenkinson and Smith, 2001).

### Resting-state functional connectivity

#### Seed description

Based on a-priori hypothesis, we created seed ROIs for the right dmPFC (18 34 29) and the left dmPFC (− 24 35 28). These areas have been shown to have increased connectivity within the default mode network and the affective network in the recent resting-state functional connectivity study in depressed patients by Sheline et al. (2010). We used Wake Forest University Pickatlas tool in SPM5 to create the dorsal nexus spheres of 10-mm radius. We also created seed ROIs for the right nucleus accumbens (10 10 − 8), the subgenual cingulate (2 22 − 18) and the right amygdala (24 − 4 − 18) as these areas are part of the affective network and have been shown to be modulated by the action of antidepressant drugs in our previous studies (McCabe et al., 2010; Norbury et al., 2007). Further these areas are specifically implicated in fMRI studies in depression (Botteron et al., 2002; Drevets et al., 2002, 1997; Sheline et al., 2001). For the subgenual cingulate cortex we used Wake Forest University Pickatlas tool in SPM5 to create the sphere of 10-mm radius. The nucleus accumbens and the amygdala spheres were created with the Harvard-Oxford subcortical structural atlas probabilistic atlas (Kennedy et al., 1998; Makris et al., 1999) so as to maximise the exact coverage of these smaller differentially shaped structures, furthermore the nucleus accumbens and the amygdala masks were thresholded by 20%.

### Time series extraction and higher-level analysis

**Fig. 1:**
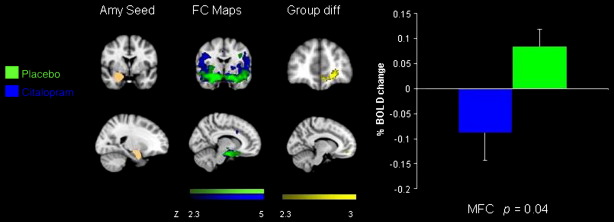
Coronal and sagittal slices showing amygdala seed region, functional connectivity maps for the placebo and citalopram groups, the group difference in connectivity with the seed region and a graph of the % BOLD signal change extracted from the cluster of significant difference between the groups [vmPFC − 6 46 − 10 *p* = 0.04].

**Fig. 2:**
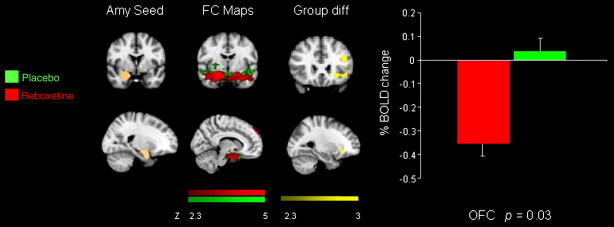
Coronal and sagittal slices showing amygdala seed region, functional connectivity maps for the placebo and reboxetine groups, the group difference in connectivity with the seed region and a graph of the % BOLD signal change extracted from the cluster of significant difference between the groups [OFC − 26 28 − 8 *p* = 0.0002]. Bars indicate SEM.

**Fig. 3:**
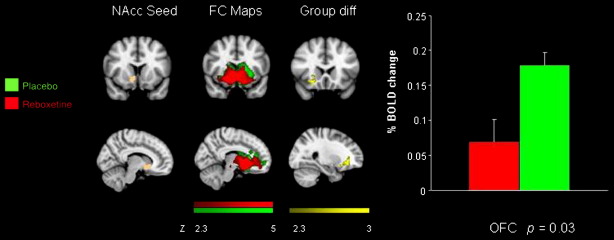
Coronal and sagittal slices showing nucleus accumbens seed region, functional connectivity maps for the placebo and reboxetine groups, the group difference in connectivity with the seed region and a graph of the % BOLD signal change extracted from the cluster of significant difference between the groups [OFC 26 20 − 12 *p* = 0.03].

An FSL tool (Featquery) was used to extract the time series within each of the five seed regions for each individual subject after having pre-processed the raw fMRI data. Time series were averaged across all voxels in each seed ROI. Using the extracted time series we performed multiple regression analysis using FMRI data processing FEAT. These analyses produced separate individual participant-level correlation maps of all voxels that were positively or negatively correlated with each of the five seeds. Afterwards, higher level (group level) analysis was carried out using FMRIB's Local Analysis of Mixed Effects (FLAME) (Woolrich et al., 2004). The general linear model (GLM) was applied to test for group averages and differences among the three groups (citalopram, reboxetine and placebo). The *Z* statistic images were thresholded using clusters determined by *Z* > 2.3, and a whole brain family-wise error-corrected cluster significance threshold of *p* < 0.05 was applied to the superthreshold clusters. Graphs of % BOLD signal change (Figs. 1–3) were created with the FSl tool, Featquery (www.fmrib.ox.ac.uk/fsl) (Smith et al., 2004).

### Nuisance signal regression

In order to account for potential indeterminate noise, nine covariates of no interest (nuisance) were identified for inclusion in our analyses. In detail, global signal, white matter (WM), cerebrospinal fluid (CSF), and the 6 motion parameters for each individual were added. As the global signal is thought to reflect a combination of physiological processes (such as cardiac and respiratory fluctuations) and scanner drift, it was included as a nuisance signal to minimise the influence of such factors (Birn et al., 2006; Gavrilescu et al., 2002; Macey et al., 2004). In order to extract the nuisance covariate time series for WM and CSF, we firstly segmented each individual's high-resolution structural image, using FSL's FAST segmentation program. The resulting segmented WM and CSF images were then thresholded to ensure 80% tissue type probability. These thresholded masks were then applied to each individual's time series, and a mean time series was calculated by averaging across all voxels within the mask (Fox et al., 2005).

## Results

### Demographic details and mood ratings

There were no significant differences among the three groups as determined by one-way analyses of variance (ANOVAs) for age, gender or body mass index, *p* > 0.06 (Table 1). There were no significant differences between the three groups as determined by one-way ANOVAs for measures of anhedonia (Snaith–Hamilton Pleasure Scale, Fawcett–Clarke Pleasure Scale) trait anxiety (TRAIT) or mood (Beck Depression Inventory), *p* > 0.1 (Table 1). Treatment with reboxetine or citalopram also failed to affect subjective state and mood measured over the 7-day experimental period on visual analogue scales (alertness, disgust, drowsiness, anxiety, happiness, nausea, and sadness) as determined by repeated-measures ANOVAs, *p* > 0.1 (Table S1 in Supplement 1).

### Functional connectivity: placebo

For each of the five seed regions previously associated with depression and the effects of antidepressant medications on neural processing, seed-based correlation analyses were employed to characterise their associated functional systems during rest. The functional connectivity for the five seed regions is described in Supplementary Table 2 for the placebo group alone (baseline). Overall, the patterns of connectivity associated with each of the seed regions are consistent with other neuroimaging studies examining the correlations between these seed regions and other brain areas in resting-state and functional connectivity experiments in healthy controls and depressed patients (Anand et al., 2005b; Bluhm et al., 2009; Fox and Raichle, 2007; Frodl et al., 2010; Greicius et al., 2007; Raichle et al., 2001; Raichle and Snyder, 2007; Zhou et al. 2010).

### Functional connectivity: drug vs. placebo

#### Amygdala seed

Compared to the placebo group there was reduced functional connectivity between the amygdala and the ventral medial prefrontal cortex (vmPFC) in the citalopram group compared to placebo (Fig. 1). There was also reduced functional connectivity between the amygdala and the OFC in the reboxetine group compared to placebo (Fig. 2) (Table 2).

#### Nucleus accumbens seed

Compared to the placebo group there was reduced functional connectivity between the nucleus accumbens and the mid OFC in the reboxetine group (Fig. 3). There were no differences between the placebo and the citalopram group for this seed region (Table 2).

#### dmPFC seed

There were no differences between the placebo and the reboxetine group for this seed region. Compared to the placebo group there was reduced functional connectivity between the dmPFC seed region and the hippocampus in the citalopram group (previously published McCabe et al., 2011).

#### Subgenual seed

There were no differences between the placebo group and either of the drug groups citalopram or reboxetine in subgenual functional connectivity.

## Discussion

Our study reveals that 7 day administration with the antidepressants citalopram and reboxetine modulates resting-state functional connectivity in a double-blind placebo controlled design in healthy volunteers. Specifically, resting-state functional connectivity was reduced between the prefrontal cortex and the amygdala for both the reboxetine and the citalopram groups. Further there was reduced connectivity between the OFC and the striatum in the reboxetine group compared to the placebo group. Consistently previous studies using task related functional MRI have also shown these limbic areas to be modulated by citalopram and reboxetine (McCabe et al., 2010) and previous studies of resting-state functional connectivity in depressed patients reveal dysfunction in these regions (Anand et al., 2005a; Cullen et al., 2009; Greicius et al., 2007; Zhou et al., 2010). Unlike our previous finding of reduced dmPFC connectivity with the hippocampus in the citalopram group compared to placebo (McCabe et al., 2011) we found no effects on connectivity with the dmPFC for the reboxetine group. We also found no effects of the antidepressants on connectivity with the subgenual cingulate cortex.

In our study both antidepressants reduced connectivity between the amygdala and parts of the prefrontal cortex. Such effects are potentially relevant to the treatment of depression as a number of task related studies have revealed reduced amygdala activity after antidepressant drug treatment (Harmer et al., 2006; Murphy et al., 2009; Norbury et al., 2007; Sheline et al., 2001) in healthy and depressed volunteers. Furthermore a recent study by Versace et al. (2010) revealed that depressed and bipolar patients were associated with increased amygdala-orbitofrontal cortex connectivity compared to healthy controls. Also the patients currently treated with antidepressants had reduced functional connectivity in these areas (Versace et al., 2010) and the authors concluded that the increased connectivity may serve as a state marker for depression. Our results indicate that using a randomised placebo controlled design we were able to extend these findings to resting-state functional connectivity in healthy volunteers with antidepressant treatment and we suggest that the normalisation of connectivity could be the direct result of the pharmacological treatment.

We also found that the connectivity between the OFC and the striatum was reduced with reboxetine treatment during our resting-state scan. The striatum has been implicated previously in studies on resting-state functional connectivity in depression (Bluhm et al., 2009; Greicius et al., 2007) and a recent study in depressed patients found that there was increased functional connectivity in the striatum, the amygdala and the hippocampus during negative emotional processing (Hamilton and Gotlib, 2008). The authors concluded that depressed individuals over-recruit neural networks involved more generally in enhancing memory for affective stimuli and that the degree to which they over-recruit this system is related to the severity of clinical symptomatology (Hamilton and Gotlib, 2008). Our results are the first to show that these areas which are known to be involved in the affective salience of stimuli, the representation of reward value and self-referential processing can be modulated by currently used antidepressant medications outside of a task situation and in unmedicated healthy control volunteers.

The OFC/vmPFC has been described as the principal mediator in a reciprocal relationship between the subcortical–cortical regions in depression (Seminowicz et al., 2004) and a recent study has revealed aberrant mutual excitation between the medial prefrontal cortex and part of the ventral anterior cingulate in depressed patients which also positively correlated with increased scores on a depressive rumination scale (Hamilton et al., 2010). This region is very close to the region of vmPFC that we have found to have reduced connectivity with the amygdala in the citalopram group (Table 2). As the medial PFC has been reported to underlie the evaluation of the self (Gusnard et al., 2001; Northoff and Bermpohl, 2004) it has been hypothesised that over activity in this region might be responsible for the maladaptive rumination seen in depression (Hamilton et al., 2010). Furthermore a recent study by Frodl et al. (2010) discusses how the integrative function of the OFC with its convergence of connections from the subcortical limbic regions is a key player in the pathophysiology of major depression (Frodl et al., 2010). Disrupted connectivity between the vmPFC/OFC and regions outlined here such as the amygdala and the striatum may thus underlay the aberrant negative and positive emotional processing witnessed in depression. Specifically related to the recent Sheline et al. (2010) study on resting-state functional connectivity in depressed patients we have previously examined the dmPFC (dorsal nexus) as a seed region and found that the antidepressant citalopram reduced the connectivity between the dmPFC and a part of the default mode network the hippocampus in our healthy volunteers, we concluded that antidepressant medications may work by reducing the aberrant increased connectivity seen in depression (McCabe et al., 2011). Interestingly though in this study we did not find any effects of reboxetine on dmPFC connectivity. This may simply be due to power and perhaps with increased numbers of volunteers differences may have been revealed. Or it could also be due to the relatively less efficacious nature of reboxetine as a treatment for depression (Cipriani et al., 2009; Eyding et al., 2010). Although we ran our resting-state approximately 10–15 min after a task it is possible that examining resting-state after other tasks could effect the results and thus we intend for future experiments to run resting-state scans before any other tasks.

As hypothesised reboxetine decreased mesolimbic striatal–OFC connectivity (indicating catecholaminergic pathway involvement) whereas citalopram decreased amygdala-vmPFC connectivity ((and in our previous study hippocampal–dmPFC connectivity) indicating serotonergic pathway involvement). These results are consistent with recent studies in animals using pharmacological challenges to unpick rat brain connectivity. Schwarz and colleagues examined pharmacological challenges such as amphetamine (catecholamine) and fluoxetine (SSRI) on rat brain functional connectivity. Similarly they report that catecholaminergic drugs modulate striatal and prefrontal connections whereas serotonergic drugs modulate hippocampal, amygdala and thalamus connections (Schwarz et al., 2009; Schwarz et al., 2007a,b). Yet this is the first examination, to the best of our knowledge, of the effects of SSRI and SNRI antidepressant drug action in the resting human brain. Similar to Schwarz et al. we also found that the regions affected by the drugs closely reflect the known pathways in the neurotransmitter systems targeted by these drugs. Further work on receptor mapping would of course also aid our understanding of the brains architecture and would be especially relevant for pharmacological studies such as this (Zilles and Amunts, 2009).

There was no effect of either of the antidepressant medications on connectivity with the subgenual cingulate cortex seed, despite reports of increased subgenual connectivity in depressed patients resting-state data (Sheline et al., 2010). The subgenual cingulate cortex is an important area targeted in deep brain stimulation for treatment-resistant depression (Kennedy et al., 2011; Lozano et al., 2008; Mayberg et al., 2005) perhaps then antidepressant improvement may lie in the development of drugs that can better target regions such as the subgenual cingulate cortex as well as the dmPFC (Sheline et al., 2010). The subgenual cingulate is also a region that is hard to image and thus it cannot be ruled out that our results might in some way reflect signal drop-out in this area.

In conclusion, the results from this study show that both serotonergic and noradrenergic antidepressants decrease resting-state functional connectivity in areas of the brain that correspond with the central activity of these drugs. The results also support our hypothesis that antidepressant medications may work by rebalancing cortical control in depression. It will be of great interest to examine resting-state functional connectivity in unmedicated depressed patients before and after antidepressant treatment as the next step in unravelling antidepressant drug action at the neural level.

## Figures and Tables

**Table 1:** Group demographic and psychosocial measures.

Measure	Citalopram	Reboxetine	Placebo
	(*n* = 12)	(*n* = 13)	(*n* = 15)
	Mean (s.d.)	Mean (s.d.)	Mean (s.d.)
Age, years	25.5 (5)	26 (3.8)	25.2 (5)
Gender	M = 6, F = 6	M = 5, F = 8	M = 7, F = 8
BDI	2.8 (3.4)	1.3(1.8)	1.2 (1.2)
TRAIT	34 (5.9)	32.6 (7.6)	31.7 (4.8)
FCPS	126 (13)	140 (14)	136.6 (14)
SHAPS	23.7 (4)	22 (4.8)	19.9(5.7)
BMI	24.4(3.5)	21.8(2.3)	24(2.8)

BDI, Beck Depression Inventory; FCPS, Fawcett–Clarke Pleasure Scale; SHAPS, Snaith–Hamilton Pleasure Scale; TRAIT, State-Trait anxiety inventory; BMI, Body Mass Index.

One-way ANOVAs all *p* > 0.06.

**Table 2:** Regions showing significant effect of treatment on connectivity with seed regions relative to placebo.

Brain region	Montreal Neurological Institute (MNI) coordinates			*Z*-score	Significance (*p*-value)
*X*	*Y*	*Z*
**NAcc seed [10, 10, − 8]**					
*Placebo > Reboxetine*					
OFC (R)	26	20	− 12	3.84	0.03

**Amygdala seed [24, − 4, − 18]**					
*Placebo > Citalopram*					
vmPFC (L)	− 6	46	− 10	2.85	0.04

*Placebo > Reboxetine*					
OFC (L)	− 28	26	− 8	3.66	0.0002
LOFC (L)	− 44	20	− 6	3.66	0.0002

R, right; L, left; vmPFC, ventral medial prefrontal cortex; LOFC, lateral orbitofrontal cortex; OFC, orbitofrontal cortex; NAcc, Nucleus accumbens; *p*-values clusters whole brain fully corrected (FWE *p* < 0.05).

## References

[bb0005] Anand A., Li Y., Wang Y., Wu J., Gao S., Bukhari L., Mathews V.P., Kalnin A., Lowe M.J. (2005). Activity and connectivity of brain mood regulating circuit in depression: a functional magnetic resonance study. Biol. Psychiatry.

[bb0010] Anand A., Li Y., Wang Y., Wu J., Gao S., Bukhari L., Mathews V.P., Kalnin A., Lowe M.J. (2005). Antidepressant effect on connectivity of the mood-regulating circuit: an FMRI study. Neuropsychopharmacology.

[bb0015] Beck A.T., Ward C.H., Mendelson M., Mock J., Erbaugh J. (1961). An inventory for measuring depression. Arch. Gen. Psychiatry.

[bb0020] Birn R.M., Diamond J.B., Smith M.A., Bandettini P.A. (2006). Separating respiratory-variation-related fluctuations from neuronal-activity-related fluctuations in fMRI. NeuroImage.

[bb0025] Bluhm R., Williamson P., Lanius R., Theberge J., Densmore M., Bartha R., Neufeld R., Osuch E. (2009). Resting state default-mode network connectivity in early depression using a seed region-of-interest analysis: decreased connectivity with caudate nucleus. Psychiatry Clin. Neurosci..

[bb0030] Botteron K.N., Raichle M.E., Drevets W.C., Heath A.C., Todd R.D. (2002). Volumetric reduction in left subgenual prefrontal cortex in early onset depression. Biol. Psychiatry.

[bb0035] Buckner R.L., Andrews-Hanna J.R., Schacter D.L. (2008). The brain's default network: anatomy, function, and relevance to disease. Ann. N. Y. Acad. Sci..

[bb0040] Cipriani A., Furukawa T.A., Salanti G., Geddes J.R., Higgins J.P., Churchill R., Watanabe N., Nakagawa A., Omori I.M., McGuire H., Tansella M., Barbui C. (2009). Comparative efficacy and acceptability of 12 new-generation antidepressants: a multiple-treatments meta-analysis. Lancet.

[bb0045] Cullen K.R., Gee D.G., Klimes-Dougan B., Gabbay V., Hulvershorn L., Mueller B.A., Camchong J., Bell C.J., Houri A., Kumra S., Lim K.O., Castellanos F.X., Milham M.P. (2009). A preliminary study of functional connectivity in comorbid adolescent depression. Neurosci. Lett..

[bb0050] Davidson R.J., Pizzagalli D., Nitschke J.B., Putnam K. (2002). Depression: perspectives from affective neuroscience. Annu. Rev. Psychol..

[bb0055] DeRubeis R.J., Hollon S.D., Amsterdam J.D., Shelton R.C., Young P.R., Salomon R.M., O'Reardon J.P., Lovett M.L., Gladis M.M., Brown L.L., Gallop R. (2005). Cognitive therapy vs medications in the treatment of moderate to severe depression. Arch. Gen. Psychiatry.

[bb0060] Drevets W.C., Price J.L., Simpson J.R., Todd R.D., Reich T., Vannier M., Raichle M.E. (1997). Subgenual prefrontal cortex abnormalities in mood disorders. Nature.

[bb0065] Drevets W.C., Bogers W., Raichle M.E. (2002). Functional anatomical correlates of antidepressant drug treatment assessed using PET measures of regional glucose metabolism. Eur. Neuropsychopharmacol..

[bb0070] Eshel N., Roiser J.P. (2010). Reward and punishment processing in depression. Biol. Psychiatry.

[bb0075] Eyding D., Lelgemann M., Grouven U., Harter M., Kromp M., Kaiser T., Kerekes M.F., Gerken M., Wieseler B. (2010). Reboxetine for acute treatment of major depression: systematic review and meta-analysis of published and unpublished placebo and selective serotonin reuptake inhibitor controlled trials. BMJ.

[bb0085] Fawcett J., Clark D.C., Scheftner W.A., Gibbons R.D. (1983). Assessing anhedonia in psychiatric patients. Arch. Gen. Psychiatry.

[bb0090] Fitzgerald P.B., Oxley T.J., Laird A.R., Kulkarni J., Egan G.F., Daskalakis Z.J. (2006). An analysis of functional neuroimaging studies of dorsolateral prefrontal cortical activity in depression. Psychiatry Res..

[bb0095] Fox M.D., Raichle M.E. (2007). Spontaneous fluctuations in brain activity observed with functional magnetic resonance imaging. Nat. Rev. Neurosci..

[bb0100] Fox M.D., Snyder A.Z., Vincent J.L., Corbetta M., Van Essen D.C., Raichle M.E. (2005). The human brain is intrinsically organized into dynamic, anticorrelated functional networks. Proc. Natl. Acad. Sci. U. S. A..

[bb0105] Frodl T., Bokde A.L., Scheuerecker J., Lisiecka D., Schoepf V., Hampel H., Moller H.J., Bruckmann H., Wiesmann M., Meisenzahl E. (2010). Functional connectivity bias of the orbitofrontal cortex in drug-free patients with major depression. Biol. Psychiatry.

[bb0110] Fu C.H., Williams S.C., Cleare A.J., Brammer M.J., Walsh N.D., Kim J., Andrew C.M., Pich E.M., Williams P.M., Reed L.J., Mitterschiffthaler M.T., Suckling J., Bullmore E.T. (2004). Attenuation of the neural response to sad faces in major depression by antidepressant treatment: a prospective, event-related functional magnetic resonance imaging study. Arch. Gen. Psychiatry.

[bb0115] Gavrilescu M., Shaw M.E., Stuart G.W., Eckersley P., Svalbe I.D., Egan G.F. (2002). Simulation of the effects of global normalization procedures in functional MRI. NeuroImage.

[bb0120] Greicius M.D., Flores B.H., Menon V., Glover G.H., Solvason H.B., Kenna H., Reiss A.L., Schatzberg A.F. (2007). Resting-state functional connectivity in major depression: abnormally increased contributions from subgenual cingulate cortex and thalamus. Biol. Psychiatry.

[bb0125] Grimm S., Ernst J., Boesiger P., Schuepbach D., Boeker H., Northoff G. (2011). Reduced negative BOLD responses in the default-mode network and increased self-focus in depression. World J. Biol. Psychiatry.

[bb0130] Gusnard D.A., Akbudak E., Shulman G.L., Raichle M.E. (2001). Medial prefrontal cortex and self-referential mental activity: relation to a default mode of brain function. Proc. Natl. Acad. Sci. U. S. A..

[bb0135] Hamilton J.P., Gotlib I.H. (2008). Neural substrates of increased memory sensitivity for negative stimuli in major depression. Biol. Psychiatry.

[bb0140] Hamilton J.P., Chen G., Thomason M.E., Schwartz M.E., Gotlib I.H. (2010). Investigating neural primacy in major depressive disorder: multivariate Granger causality analysis of resting-state fMRI time-series data. Mol. Psychiatry.

[bb0145] Harmer C.J., Shelley N.C., Cowen P.J., Goodwin G.M. (2004). Increased positive versus negative affective perception and memory in healthy volunteers following selective serotonin and norepinephrine reuptake inhibition. Am. J. Psychiatry.

[bb0150] Harmer C.J., Mackay C.E., Reid C.B., Cowen P.J., Goodwin G.M. (2006). Antidepressant drug treatment modifies the neural processing of nonconscious threat cues. Biol. Psychiatry.

[bb0155] Harmer C.J., Goodwin G.M., Cowen P.J. (2009). Why do antidepressants take so long to work? A cognitive neuropsychological model of antidepressant drug action. Br. J. Psychiatry.

[bb0160] Hollon S.D., DeRubeis R.J., Shelton R.C., Amsterdam J.D., Salomon R.M., O'Reardon J.P., Lovett M.L., Young P.R., Haman K.L., Freeman B.B., Gallop R. (2005). Prevention of relapse following cognitive therapy vs medications in moderate to severe depression. Arch. Gen. Psychiatry.

[bb0165] Jenkinson M., Smith S. (2001). A global optimisation method for robust affine registration of brain images. Med. Image Anal..

[bb0170] Jenkinson M., Bannister P., Brady M., Smith S. (2002). Improved optimization for the robust and accurate linear registration and motion correction of brain images. NeuroImage.

[bb0175] Johansen-Berg H., Gutman D.A., Behrens T.E., Matthews P.M., Rushworth M.F., Katz E., Lozano A.M., Mayberg H.S. (2008). Anatomical connectivity of the subgenual cingulate region targeted with deep brain stimulation for treatment-resistant depression. Cereb. Cortex.

[bb0180] Kennedy D.N., Lange N., Makris N., Bates J., Meyer J., Caviness V.S. (1998). Gyri of the human neocortex: an MRI-based analysis of volume and variance. Cereb. Cortex.

[bb0185] Kennedy S.H., Giacobbe P., Rizvi S.J., Placenza F.M., Nishikawa Y., Mayberg H.S., Lozano A.M. (2011). Deep brain stimulation for treatment-resistant depression: follow-up after 3 to 6 years. Am. J. Psychiatry.

[bb0190] Lisiecka D., Meisenzahl E., Scheuerecker J., Schoepf V., Whitty P., Chaney A., Moeller H.J., Wiesmann M., Frodl T. (2011). Neural correlates of treatment outcome in major depression. Int. J. Neuropsychopharmacol..

[bb0195] Lozano A.M., Mayberg H.S., Giacobbe P., Hamani C., Craddock R.C., Kennedy S.H. (2008). Subcallosal cingulate gyrus deep brain stimulation for treatment-resistant depression. Biol. Psychiatry.

[bb0200] Macey P.M., Macey K.E., Kumar R., Harper R.M. (2004). A method for removal of global effects from fMRI time series. NeuroImage.

[bb0205] Makris N., Meyer J.W., Bates J.F., Yeterian E.H., Kennedy D.N., Caviness V.S. (1999). MRI-Based topographic parcellation of human cerebral white matter and nuclei II. Rationale and applications with systematics of cerebral connectivity. NeuroImage.

[bb0210] Mayberg H.S., Lozano A.M., Voon V., McNeely H.E., Seminowicz D., Hamani C., Schwalb J.M., Kennedy S.H. (2005). Deep brain stimulation for treatment-resistant depression. Neuron.

[bb0215] McCabe C., Mishor Z., Cowen P.J., Harmer C.J. (2010). Diminished neural processing of aversive and rewarding stimuli during selective serotonin reuptake inhibitor treatment. Biol. Psychiatry.

[bb0220] McCabe C., Mishor Z., Filippini N., Cowen P.J., Taylor M.J., Harmer C.J. (2011). SSRI administration reduces resting state functional connectivity in dorso-medial prefrontal cortex. Mol. Psychiatry..

[bb0225] Mizoguchi N., Saigusa T., Aono Y., Sekino R., Takada K., Oi Y., Ueda K., Koshikawa N., Cools A.R. (2008). The reboxetine-induced increase of accumbal dopamine efflux is inhibited by l-propranolol: a microdialysis study with freely moving rats. Eur. J. Pharmacol..

[bb0230] Murphy S.E., Norbury R., O'Sullivan U., Cowen P.J., Harmer C.J. (2009). Effect of a single dose of citalopram on amygdala response to emotional faces. Br. J. Psychiatry.

[bb0235] Norbury R., Mackay C.E., Cowen P.J., Goodwin G.M., Harmer C.J. (2007). Short-term antidepressant treatment and facial processing. Functional magnetic resonance imaging study. Br. J. Psychiatry.

[bb0240] Northoff G., Bermpohl F. (2004). Cortical midline structures and the self. Trends Cogn. Sci..

[bb0245] Northoff G., Wiebking C., Feinberg T., Panksepp J. (2010). The 'resting-state hypothesis' of major depressive disorder-A translational subcortical–cortical framework for a system disorder. Neurosci. Biobehav. Rev..

[bb0250] Price J., Drevets W.C. (2009). Neurocircuitry of mood disorders. Neuropsychopharmacol. Rev..

[bb0255] Raichle M.E., Snyder A.Z. (2007). A default mode of brain function: a brief history of an evolving idea. NeuroImage.

[bb0260] Raichle M.E., MacLeod A.M., Snyder A.Z., Powers W.J., Gusnard D.A., Shulman G.L. (2001). A default mode of brain function. Proc. Natl. Acad. Sci. U. S. A..

[bb0265] Rogers M.A., Kasai K., Koji M., Fukuda R., Iwanami A., Nakagome K., Fukuda M., Kato N. (2004). Executive and prefrontal dysfunction in unipolar depression: a review of neuropsychological and imaging evidence. Neurosci. Res..

[bb0270] Schlosser R.G., Wagner G., Koch K., Dahnke R., Reichenbach J.R., Sauer H. (2008). Fronto-cingulate effective connectivity in major depression: a study with fMRI and dynamic causal modeling. NeuroImage.

[bb0275] Schwarz A.J., Gozzi A., Reese T., Bifone A. (2007). Functional connectivity in the pharmacologically activated brain: resolving networks of correlated responses to d-amphetamine. Magn. Reson. Med..

[bb0280] Schwarz A.J., Gozzi A., Reese T., Bifone A. (2007). In vivo mapping of functional connectivity in neurotransmitter systems using pharmacological MRI. NeuroImage.

[bb0285] Schwarz A.J., Gozzi A., Bifone A. (2009). Community structure in networks of functional connectivity: resolving functional organization in the rat brain with pharmacological MRI. NeuroImage.

[bb0290] Seminowicz D.A., Mayberg H.S., McIntosh A.R., Goldapple K., Kennedy S., Segal Z., Rafi-Tari S. (2004). Limbic-frontal circuitry in major depression: a path modeling metanalysis. NeuroImage.

[bb0295] Sheline Y.I., Barch D.M., Donnelly J.M., Ollinger J.M., Snyder A.Z., Mintun M.A. (2001). Increased amygdala response to masked emotional faces in depressed subjects resolves with antidepressant treatment: an fMRI study. Biol. Psychiatry.

[bb0300] Sheline Y.I., Barch D.M., Price J.L., Rundle M.M., Vaishnavi S.N., Snyder A.Z., Mintun M.A., Wang S., Coalson R.S., Raichle M.E. (2009). The default mode network and self-referential processes in depression. Proc. Natl. Acad. Sci. U. S. A..

[bb0305] Sheline Y.I., Price J.L., Yan Z., Mintun M.A. (2010). Resting-state functional MRI in depression unmasks increased connectivity between networks via the dorsal nexus. Proc. Natl. Acad. Sci. U. S. A..

[bb0310] Siegle G.J., Thompson W., Carter C.S., Steinhauer S.R., Thase M.E. (2007). Increased amygdala and decreased dorsolateral prefrontal BOLD responses in unipolar depression: related and independent features. Biol. Psychiatry.

[bb0315] Smith S.M. (2002). Fast robust automated brain extraction. Hum. Brain Mapp..

[bb0320] Smith S.M., Jenkinson M., Woolrich M.W., Beckmann C.F., Behrens T.E., Johansen-Berg H., Bannister P.R., De Luca M., Drobnjak I., Flitney D.E., Niazy R.K., Saunders J., Vickers J., Zhang Y., De Stefano N., Brady J.M., Matthews P.M. (2004). Advances in functional and structural MR image analysis and implementation as FSL. NeuroImage.

[bb0325] Snaith R.P., Hamilton M., Morley S., Humayan A., Hargreaves D., Trigwell P. (1995). A scale for the assessment of hedonic tone the Snaith–Hamilton Pleasure Scale. Br. J. Psychiatry.

[bb0330] Spielberger C.D. (1983). Manual for the State-Trait Anxiety Inventory.

[bb0335] Spitzer R.L., Williams J.B., Gibbon M., First M.B. (2004). Structured Clinical Interview for the DSM–IV (SCID–I/P).

[bb0340] Taylor M.J., Freemantle N., Geddes J.R., Bhagwagar Z. (2006). Early onset of selective serotonin reuptake inhibitor antidepressant action: systematic review and meta-analysis. Arch. Gen. Psychiatry.

[bb0345] Valentini V., Frau R., Di Chiara G. (2004). Noradrenaline transporter blockers raise extracellular dopamine in medial prefrontal but not parietal and occipital cortex: differences with mianserin and clozapine. J. Neurochem..

[bb0350] Vasic N., Walter H., Sambataro F., Wolf R.C. (2009). Aberrant functional connectivity of dorsolateral prefrontal and cingulate networks in patients with major depression during working memory processing. Psychol. Med..

[bb0355] Versace A., Thompson W.K., Zhou D., Almeida J.R., Hassel S., Klein C.R., Kupfer D.J., Phillips M.L. (2010). Abnormal left and right amygdala-orbitofrontal cortical functional connectivity to emotional faces: state versus trait vulnerability markers of depression in bipolar disorder. Biol. Psychiatry.

[bb0360] Victor T.A., Furey M.L., Fromm S.J., Ohman A., Drevets W.C. (2010). Relationship between amygdala responses to masked faces and mood state and treatment in major depressive disorder. Arch. Gen. Psychiatry.

[bb0365] Wagner G., Koch K., Schachtzabel C., Sobanski T., Reichenbach J.R., Sauer H., Schlosser R.G. (2010). Differential effects of serotonergic and noradrenergic antidepressants on brain activity during a cognitive control task and neurofunctional prediction of treatment outcome in patients with depression. J. Psychiatry Neurosci..

[bb0370] Wilson J.L., Jenkinson M., de Araujo I., Kringelbach M.L., Rolls E.T., Jezzard P. (2002). Fast, fully automated global and local magnetic field optimization for fMRI of the human brain. NeuroImage.

[bb0375] Woolrich M.W., Behrens T.E., Beckmann C.F., Jenkinson M., Smith S.M. (2004). Multilevel linear modelling for FMRI group analysis using Bayesian inference. NeuroImage.

[bb0380] Zhou Y., Yu C., Zheng H., Liu Y., Song M., Qin W., Li K., Jiang T. (2010). Increased neural resources recruitment in the intrinsic organization in major depression. J. Affect. Disord..

[bb0390] Zilles K., Amunts K. (2009). Receptor mapping: architecture of the human cerebral cortex. Curr. Opin. Neurol..

