# Supplementary material for: Antidepressant medications reduce subcortical–cortical resting-state functional connectivity in healthy volunteers
Source: Neuroimage. 2011 Aug 15;57(4):1317–23. doi: 10.1016/j.neuroimage.2011.05.051 (PMC3141109; doi:10.1016/j.neuroimage.2011.05.051)
Supplement: Supplementary file 1 — Supplementary materials [file mmc1.doc]

**Supplementary Online Material For**

**Antidepressant Medications Reduce Subcortical-cortical Resting-State Functional Connectivity in Healthy Volunteers.**

Ciara McCabe, Zevic Mishor.

* To whom correspondence should be addressed. E-mail: [ciara.mccabe@psych.ox.ac.uk](mailto:ciara.mccabe@psych.ox.ac.uk)

**This doc includes:**

Supplementary Tables 1-2

| ***TABLE S1. Subjective state ratings before and after 7 d treatment with citalopram, reboxetine or placebo. There were no significant effects of treatment group on any of the measures.*** | | | |
| --- | --- | --- | --- |
| **Measure** | **Citalopram**  **(n=12)**  **Mean (s.d.)** | **Reboxetine**  **(n=13)**  **Mean (s.d.)** | **Placebo**  **(n=15)**  **Mean (s.d.)** |
|  | Pre-treat Post-treat | Pre-treat Post-treat | Pre-treat Post-treat |
| **Alertness** | 64.8 (8.9) 55.4(21.7) | 74.6 (10.2) 64.6 (22) | 63.8 (18) 57.5 (28) |
| **Disgust** | 13.4 (15.5) 10.8 (18.6) | 7.6 (5.6) 10.8 (26.1) | 6 (6.5) 6.7 (7.3) |
| **Drowsiness** | 34.5 (22.6) 9.7 (6.3) | 21 (20) 16.3(22) | 27.4(20) 16.8 (15.5) |
| **Anxiety** | 12 (7.5) 17.2 (17) | 13.2 (12.4) 12.3 (19.4) | 14.2 (22) 7.8 (8.8) |
| **Happiness** | 71.5 (14) 61.6 (18) | 75.3 (13) 70.8 (14) | 75.3 (12.1) 72 (13) |
| **Nausea** | 7.8 (7.2) 12.3 (10.6) | 3.4 (4.9) 12.1 (27.4) | 3.9 (2.8) 4.8 (5.6) |
| **Sadness** | 13.6 (15) 10.4(14) | 7.4 (6) 13.5 (6) | 7.7 (5.6) 7.3 (6) |
| **State Anxiety** | 32.1 (6.9) 36.8 (11) | 31.2 (10.2) 33.5 (12.9) | 31 (5.3) 28.1 (5.1) |
| Repeated measures ANOVAs all p> 0.1 | | | |

| ***TABLE S2: Regions showing positive correlation with seeds in placebo group.*** | | | | |
| --- | --- | --- | --- | --- |
| **Brain Region** | **Montreal Neurological Institute (MNI) Coordinates** | | | **Z-score** |
|  | **X** | **Y** | **Z** |  |
| **Nucleus Accumbens seed [10,10,-8]** | | | | |
| NAcc | 10 | 14 | -6 | 11.4 |
| Mid OFC | 26 | 20 | -18 | 11.4 |
| Mid OFC | -20 | 20 | -20 | 11.4 |
| L OFC | -36 | 28 | -4 | 11.4 |
| NAcc /OFC | -14 | 16 | -12 | 7.63 |
| NAcc | -6 | 12 | -10 | 6.92 |
| Caudate | -14 | 16 | -2 | 6.92 |
| Anterior cingulate | 0 | 36 | 2 | 5 |
| Paracingulate gyrus | 0 | 56 | -4 | 5 |
| **Amygdala seed [24,-4,-18]** | | | | |
| Amygdala | 20 | -4 | -18 | 9.04 |
| Amygdala | -16 | -4 | -20 | 7.13 |
| Mid OFC | 24 | 12 | -22 | 6.88 |
| Mid OFC | -22 | 2 | -22 | 6.88 |
| Parahippacampal gyrus | 26 | 4 | -18 | 6.88 |
| Brain stem | 8 | -12 | -20 | 5.33 |
| Planum Polare | 40 | -2 | -18 | 5.31 |
| Temporal pole | -34 | 4 | -22 | 5.03 |
| **Subgenual seed [2,22,-18]** | | | | |
| Subgenual cortex | 6 | 22 | -18 | 7.88 |
| Subgenual cortex | 4 | 16 | -20 | 7.73 |
| Temporal pole | 42 | 14 | -26 | 4.67 |
| Temporal pole | -44 | 18 | -20 | 4.53 |
| Mid/L OFC | 32 | 16 | -22 | 4.46 |
| Superior frontal gyrus | 18 | 28 | 36 | 4.27 |
| Middle frontal gyrus | 26 | 16 | 44 | 3.16 |
| **dmPFC seed [-24,35,28]** | | | | |
| dmPFC | -20 | 32 | 30 | 7.17 |
| Temporal gyrus | -58 | -16 | -24 | 4.09 |
| Hippocampus | -20 | -22 | -18 | 4.1 |
| LOFC | -32 | 26 | -8 | 4.24 |
| Anterior cingulate | -4 | 36 | 22 | 7.17 |
| vmPFC | -2 | 46 | -12 | 6 |
| Subgenual cing | 0 | 24 | -16 | 6 |
| Caudate | -10 | 6 | 12 | 6 |
| vmPFC | -4 | 44 | -16 | 6 |
| **dmPFC [18,34,29]** | | | | |
| dmPFC | 26 | 30 | 30 | 7.21 |
| Paracingulate gyrus | 18 | 30 | 30 | 6.51 |
| dlPFC | 30 | 28 | 38 | 5.87 |
| Frontal pole | -34 | 46 | 20 | 4.56 |
| Pregenual cingulate | -6 | 40 | -6 | 3.99 |
| Insula | 34 | 16 | 0 | 3.89 |
| NAcc nucleus accumbens; OFC orbitofrontal cortex; Mid Middle; L lateral; dmPFC dorsal medial prefrontal cortex; vmPFC ventral medial prefrontal cortex.  P<0.05 FWE whole brain corrected. | | | | |
